# Defining the passage of wisdom: A taxonomy of supervision for RANZCP trainees and Fellows

**DOI:** 10.1177/10398562241231877

**Published:** 2024-02-13

**Authors:** Edward Miller, Michael James Weightman, Andrew Amos, Stephen Parker

**Affiliations:** Division of Psychological Medicine, 1415 The University of Auckland, Auckland, New Zealand; School of Medicine, 1066 The University of Adelaide, Adelaide, SA, Australia; College of Medicine and Public Health, Flinders University, Bedford Park, SA, Australia; School of Medicine and Dentistry, 104397 James Cook University, Townsville, QLD, Australia; Faculty of Medicine, 420004 University of Queensland, Herston, QLD, Australia; Metro North Mental Health, Herston, QLD, Australia

**Keywords:** supervision, medical education, Royal Australian and New Zealand College of Psychiatrists

## Abstract

**Objective:**

Trainees and Fellows of the Royal Australian and New Zealand College of Psychiatrists (RANZCP) work in complex interpersonal and organisational environments. Engagement in supervision can be a helpful way for trainees and Fellows to achieve interpersonal, professional, and organisational success. Supervision comes in many forms depending on the stage and state of one’s career. An awareness of different supervision models is relevant to trainees’ understanding of what is expected of them and their supervisors in their work and educational contexts. This paper explores the taxonomy of supervision models available to RANZCP trainees and Fellows in Australia and New Zealand.

**Conclusion:**

Supervision is a heterogeneous concept with multiple aims, outcomes, and processes that change with ones’ stage of career.

The roles of RANZCP trainees and Fellows require navigation of complex professional, organisational, interpersonal, and clinical expectations and relationships.^1^ Adequate training, self-care, and continuing professional development (CPD) are central to maintaining professional standards. Like most medical subspecialities, supervision in psychiatry has roots in the apprenticeship model, whereby a junior trainee learns directly through teaching, observation and feedback from a more senior practitioner.^2^ In psychiatry, supervision has also been influenced by the traditional psychoanalytic dyad.^3^ The RANZCP emphasises the central role of supervision in psychiatry training and practice,^4^ however, the supervision concept has received scant research attention, and is not a unified entity with a single definition.^2^ At least six main types of supervision are now available to junior and senior RANZCP practitioners, which are summarised in Table 1. These include (i) clinical supervision, (ii) reflective practice groups, (iii) peer groups, (iv) mentoring & external supervision, (v) psychotherapy supervision, and (vi) research & scholarly project supervision. This paper will outline these main types of supervision and contrast their aims and outcomes.

**Table 1.** Different types of supervision relevant to RANZCP trainees and Fellows, and key differentiating features and aims

Type	Key differentiating features	Key aim
Clinical supervision	• Individual (primarily) but can involve others	• Develop skills and competencies central to RANZCP training
	• Based on apprenticeship model – observe, practice and discuss clinical cases	• Identify and signpost unreadiness to progress
	• Dual role of support and assessor	• The role also includes pastoral, developmental and leadership aspects
	• Mandatory part of RANZCP training	
	• Methodology and process varies greatly; high heterogeneity	
	• Short term changing 6 monthly over the length of training	
Reflective practice groups (e.g. Balint)	• Group-based	• Support trainee wellbeing
	• Psychodynamically informed	• Foster insight into underlying psychodynamic principles which may translate back to patient interactions
	• Clear methodology and process	
	• Not mandatory in RANZCP training	
	• Short to long term depending on context	
	• May have a cost involved	
	• Trainee and consultant grades	
Psychotherapy supervision	• Individual or group supervision relating to PWC training	• Identify key psychodynamic principles of the case through the lived experience of supervision; foster insight
	• Individual supervision for psychotherapy AT or practicing psychotherapists	• Focus on personal history and experience of the supervisee particularly if undertaking psychotherapy training or practice
	• Based on psychodynamic principles although heavily influenced by individual style and practice	
	• Weekly/fortnightly (PWC) increasing up to daily if psychoanalytic	
	• Medium to longer term	
	• May have cost involved	
Mentoring & external supervision	• Individual	• Aimed at being supportive
	• Informal, optional and non-intimidating	• Can have more of a focus on supervisee’s emotional experience
	• Based primarily on experience not training, although some mentor training can be acquired	• Can help with adjustment and role transition for junior trainees
	• Short to medium term	• Can be aimed at upskilling in a specific area of interest
	• Registrar or early career consultant level	
Research & scholarly project supervision	• Either individual or networked	• Relates to passing scholarly project or participating in research or academia
	• Clear aim, methodology and process	• Aim is acquiring and understanding research practices including publishing
	• Requires high level of training on supervisor’s part	• Set up research and collaborative networks
	• Can be critical and a steep learning curve but highly rewarding if done well	
	• Short or long term	
	• Registrar or consultant level	
Peer groups	• Group based; usually a mix of junior and senior colleagues of same grade	• Discuss complex clinical or workplace issues with peers
	• Meet regularly	• Multi-directional feedback
	• Counts towards CPD and ongoing learning	
	• Mix of collegiate, clinical and supporting roles	
	• Long term	
	• May have cost involved	
	• Usually consultant level	

## Clinical supervision

The RANZCP mandates that all trainees receive at least one hour per week (two hours for Stage 1 trainees) of one-on-one supervision with their primary allocated rotation supervisor, alongside a further four hours of additional clinical supervision.^5^ Clinical supervision is central to the training and assessment of trainees, yet it paints with a broad brush, and covers many different perspectives and intended outcomes.^6^ Table 2 gives a complete list of expected roles of clinical supervisors as described by the RANZCP training guide for clinical supervision.

**Table 2.** Duties of clinical supervisors (adapted from RANZCP Supervisor manual 2012)^1^

• Be familiar with core information, inclusive of the RANZCP regulations and curriculum, code of ethics, and the procedures of the competency-based fellowship program
• Understand the basic requirements of the role and be committed to education and training
• Provide initial orientation to the training program to first-year trainees at their institution
• Provide leadership and modelling
• Monitor and observe trainees with patients, peers and other medical staff regularly
• Encourage trainees to consider a patient’s support network (family and carers) as part of the patient’s treatment and recovery
• Reflect constructively upon the work presented in supervision
• Discuss the trainee’s performance with the director of training (DOT) if required
• Discuss strategies to overcome any weaknesses in performance with the trainee concerned
• Identify problems needing remediation early, and consult the DOT
• Ensure availability to participate in the trainee’s formative workplace-based assessments (WBAs) as required
• Sign off a trainee’s entrustable professional activity (EPA) only when confident the trainee can conduct an activity with distant supervision
• Be responsible for completing a trainee’s formative mid-rotation in-training assessment (ITA) form to provide feedback to the trainee
• Be responsible for completing a trainee’s summative end-of-rotation ITA report and assist the trainee in ensuring that it reaches the college within 60 days
• Be interested and supportive of the trainee
• Understand the educational aims and objectives for the specific training rotation
• Attend reliably and be available for clinical consultation
• Attend a supervisors’ peer review group three times per year, and present at one of these meetings (minimum) or at a meeting of medical staff where supervision is discussed

Good clinical supervision is said to be vital to protect trainee burnout, particularly early in training.^7^ However, the practice of clinical supervision is highly variable, often with unclear goals based on limited evidence of effectiveness.^8^ Clinical supervision is also poorly understood, particularly by trainees.^9^ One survey found supervision objectives were not often clearly defined at the beginning of the rotation and that lack of structure, set times, flexibility, and frequent supervisor changes were barriers to a safe and supportive learning environment.^10^ Another 2012 survey of trainees found that less than half thought their clinical supervisors were supportive of trainee’s medical student teaching role and did not discuss their teaching in supervision.^11^

The change in the RANZCP’s assessment model in 2012 further shifted the focus of supervision to assessment over supportive or mentoring elements.^12^ Proposed changes to the assessment model from 2024 are likely to continue this trend, as they replace high-stakes exams with workplace-based assessments as the basis of decisions about training progression.^13^ This will present clinical supervisors with an even more complex set of competing responsibilities. These include professional development aspects such as fostering resilience, wellbeing, and an understanding of professional boundaries, to facilitating an understanding of therapeutic relationships and psychodynamic aspects of the work, whilst ensuring safe and effective clinical teaching and training, alongside additional assessment and pastoral, developmental, and leadership roles.^6^ A potential workaround to this dilemma can be found in the Royal College of Psychiatrists (RCPsych) training in the United Kingdom (UK), where an Educational Supervisor (ES) is appointed separate from the clinical supervisor in order to externalise the monitoring of a trainee’s educational progress.^14^

## Reflective practice groups

Reflective practice groups, the most common of which are called Balint groups, refer to groups where practitioners participate in non-judgemental, clinical reflective practice facilitated by a trained supervisor.^12^ Weekly Balint groups are a mandatory part of RCPsych training in the UK, but not the RANZCP training program. However, many local RANZCP training branches offer them, and they can also be privately arranged as ongoing CPD for psychiatrists.^15^ Groups meet between weekly and monthly and usually have around five to ten people. Balint groups typically centre around a participant presenting an account of a perplexing or troubling patient encounter, which elicited a strong emotional reaction in the practitioner.^3^ The other group participants then consider the emotional experience of both doctor and patient without referring to technical explanations or giving advice. The aim of this process is for participants to explore dynamics which might be involved in the interaction, and which may also play out in the group, such as parallel processes, transference and countertransference reactions, and various levels of identification and projective identification.^15,16^ Balint groups are intended to help practitioners understand their own and patient experiences, as well as deepen the understanding of complex psychodynamic aspects of cases, and may help assist with practitioner emotional well-being and preventing burnout.^12,15^

## Peer groups

Another type of group supervision is a peer group. These usually meet between weekly or monthly, and consist of 5–10 psychiatrists of differing career stages, sometimes including senior trainees. As well as contributing to CPD, a peer group offers perspective sharing between professional equals in exploring complex cases, management decisions, and current trends in research or practice. These groups also provide a supportive and encouraging environment from others who understand the complexities and vicissitudes of the role. Peer groups can also focus on a theme to provide a forum for advice and reflection for psychiatrists working in a specific area of interest (such as clinical supervisors, private practice, or veterans’ mental health).

## Mentoring & external supervision

Mentors and external supervisors both offer individual supervision. Mentors are generally referred to as teachers, advisors, or career coaches who are good at listening and talking in confidence with a mentee.^17^ Usually, mentors and mentees meet fortnightly to monthly, sometimes when more professional guidance is needed, and at other times less frequently.^18^ Common subjects are training, managing work-life balance, issues with supervision, career goals and plans, and suggestions about professional development opportunities.^18^ Affirming the mentee’s strengths and interests and providing encouragement are also helpful. There is limited research evidence on the effectiveness of mentoring but it may help with burnout, and is thought to be particularly effective for senior trainees planning for the transition to Fellowship.^19,17^

Barriers to effective mentoring include the mentor being too critical, trying to solve mentees’ problems for them, interference with the psychodynamics of rotation supervision through processes such as splitting, and the blurring of boundaries between the professional, social, and other relationships of mentors and mentees.^18^ Boundaries can be particularly challenging to negotiate in regional and rural practice. Mentoring may be individually or systemically organised – the RANZCP offers a mentoring program that gives both mentees and mentor participants programmed guidance for effective mentoring, as well as access to mentors from an area separate to their local organisation.^20^

External supervision refers to a trainee or psychiatrist regularly seeing a senior colleague outside of their workplace for support and guidance with complex clinical, organisational, or workplace issues^21^ or upskilling in a specific area. Given the seniority difference, the role includes a mentoring and supportive aspect, and may focus more on the supervisee’s emotional experience of their role, alongside facilitating career choices and direction.

## Personal psychotherapy & psychotherapy supervision

As a RANZCP trainee, the psychotherapy written case (PWC) forms a significant aspect of assessment.^4^ Supervision is most commonly conducted on an individual basis, but can also occur in groups of up to five trainees with three co-supervisors. Group supervision is more common in regional locations and has been deemed effective by trainees.^22^ Psychotherapy skills training in psychiatry has also traditionally followed an apprenticeship model, whereby trainees develop competency by practicing and discussing the practice of psychotherapy with a supervisor, with minimal direct observation.^23^ Many training sites cannot consistently provide access to accredited psychotherapy supervisors from within the workplace setting which leads to trainees seeking external supervisors.^23^

Trainees undertaking advanced training in psychotherapy require ongoing psychotherapy supervision, the frequency and length of which is dependent on the modality of psychotherapy training undertaken, and involves discussing and formulating aspects of the case based on the psychotherapeutic principles being taught.^24^ The RANZCP does not mandate private personal psychotherapy at any career grade but some professionals who choose to do this may find it beneficial, and requires another ongoing time and cost commitment.^25^

## Research and scholarly project supervision

Most research supervisors for the scholarly project are likely to be the trainee’s primary clinical supervisor.^26^ However, seeking primary or secondary supervision from a university department of psychiatry can achieve greater networking and career development benefits. A trainee should actively seek out a supervisor who meets their research interests, including compatibility between both personality and professional approaches.^26^ At the early career and trainee stage, academic supervision usually falls into role of a mentoring relationship.^26^ Academic supervision can involve discussing mutual aims and goals of the research, including a timeframe and expected outcomes and publications, and assistance with learning research skills such as formulating research questions, choosing methodological approaches, ethics applications, data collection and analysis, and giving and receiving feedback on the write-up.^27^ It may be helpful to put together a written supervisory agreement and to discuss potential boundary issues from the outset of supervision.^26^

## Other types of supervision

There are several other additional types of supervision that may be encountered, which are summarised in Table 3 in the supplementary material for completeness.

## Conclusion

Though psychiatric supervision has continuously evolved, its roots are still principally grounded in the apprenticeship model. There are now more than six main types of supervision modalities relevant to RANZCP trainees and Fellows, with the clinical supervisor role remaining central but having competing aims. Changes to the RANZCP examination process are expected to significantly impact the clinical supervisor role, and may require supportive elements to be delivered through other modalities, such as mandated Balint groups or mentors, or considering splitting the role in a similar way to the ES position in the RCPsych program. Clinical supervisors may also benefit from additional specific training to help them maintain workforce needs and supportive working relationships whilst ensuring adequate assessment standards and critical rigour across different regions. This paper has summarised the aims and features of the current mix of supervision modalities available to RANZCP trainees and Fellows, offering an overview that may help make the process easier to understand and navigate.

## Supplemental Material

Supplemental Material - Defining the passage of wisdom: A taxonomy of supervision for RANZCP trainees and Fellows Supplemental Material for Defining the passage of wisdom: A taxonomy of supervision for RANZCP trainees and Fellows by Edward Miller, Michael James Weightman, Andrew Amos, and Stephen Parker in Australasian Psychiatry.
