# Supplementary material for: Defining the passage of wisdom: A taxonomy of supervision for RANZCP trainees and Fellows
Source: Australas Psychiatry. 2024 Feb 13;32(3):242–6. doi: 10.1177/10398562241231877 (PMC11103896; doi:10.1177/10398562241231877)
Supplement: Supplemental Material - Defining the passage of wisdom: A taxonomy of supervision for RANZCP trainees and Fellows [file sj-pdf-1-apy-10.1177_10398562241231877.pdf]

Table 3: Other types of supervision

Type	Description
Remote supervision	Unique to rural and remote workers who cannot access various kinds of in person contact due to distance and workforce limitations.<br><br>Conducted over teleconference or video link, which has some advantages and disadvantages, such as being able to access bespoke psychotherapy supervisors, but may require adaptation to use [1].
Structured supervision	A form of group supervision offered by some District Health Boards (DHBs) in New Zealand.<br><br>Trainees are offered to meet with either a single or rotating supervisor weekly to discuss systemic and structural issues relating to their practice – e.g. on call and after hours issues, mental health act and other system processes [2].
Specialist International Medical Graduates (SIMG) supervision	SIMG candidates have additional supervision needs which are listed in the RANZCP Committee for SIMG Education guidelines [3].
Service-user supervision	A proposed set up where additional supervision is provided by a service-user and is aimed at helping trainees develop a more recovery-focused perspective [4].
Educational supervisor (ES)	A role in the RCPsych training pathway that is separate from the clinical supervisor role of the RANZCP.<br><br>Aims to externalise the support and monitor educational progress as separate from the clinical supervision and assessment role.<br><br>The ES works with individual trainees to facilitate an individual learning plan to develop curriculum competencies, and is responsible for up to four trainees, meeting 4-6 times per year with each one [5].

1. Coleman M, Amos A, Hoimes J, et al. Remote supervision in psychiatry training: Unlocking capacity and technology. *Australasian Psychiatry* 2022; 30: 768-770. DOI: 10.1177/10398562221127825.
2. Program NRPT. Supervision, https://www.psychtraining.org/SV2.html (2023, accessed 24.12.2023).
3. RANZCP. *Committee for Specialist International Medical Graduate Education (CSIMGE) Supervisor Guide*. 2015. Melbourne, Australia: RANZCP.
4. Bhagavan C, Gordon S and Sundram F. From the chemical imbalance to the power imbalance: A psychiatry trainee's perspectives on service-user supervision. *Australasian Psychiatry* 2023; 31: 700-704. DOI: 10.1177/10398562231191695.
5. RCPsych. RCPsych Guidance: Recognition and Approval of Trainers, https://www.rcpsych.ac.uk/docs/default-source/training/letb/rcpsych-guidance-for-recognition-of-trainers.pdf?sfvrsn=ff0fa8c4_2 (2023, accessed 24.12.2023).
